# Pesticide tolerance in amphibians: induced tolerance in susceptible populations, constitutive tolerance in tolerant populations

**DOI:** 10.1111/eva.12083

**Published:** 2013-07-25

**Authors:** Jessica Hua, Nathan I Morehouse, Rick Relyea

**Affiliations:** Department of Biological Sciences, University of PittsburghPittsburgh, PA, USA

**Keywords:** acetylcholine-inhibitor, amphibians, Ellman assay, insecticide tolerance, phenotypic plasticity

## Abstract

The role of plasticity in shaping adaptations is important to understanding the expression of traits within individuals and the evolution of populations. With increasing human impacts on the environment, one challenge is to consider how plasticity shapes responses to anthropogenic stressors such as contaminants. To our knowledge, only one study (using mosquitoes) has considered the possibility of induced insecticide tolerance. Using populations of wood frogs (*Lithobates sylvaticus*) located close to and far from agricultural fields, we discovered that exposing some populations of embryos and hatchlings to sublethal concentrations of the insecticide carbaryl induced higher tolerance to a subsequent lethal concentration later in life. Interestingly, the inducible populations were located >800 m from agricultural areas and were the most susceptible to the insecticide. In contrast, the noninducible populations were located close to agricultural areas and were the least susceptible. We also found that sublethal concentrations of carbaryl induced higher tadpole AChE concentrations in several cases. This is the first study to demonstrate inducible tolerance in a vertebrate species and the pattern of inducible and constitutive tolerance among populations suggests the process of genetic assimilation.

## Introduction

Phenotypic plasticity, which is the capacity of a single genotype to exhibit variable phenotypes in different environments, allows adaptive traits to be induced within a single generation (Schlichting and Pigliucci 1998). In rapidly changing environmental conditions, the contribution of plasticity to the process of organismal adaptation has critical implications for the expression of traits within individuals and the evolution of populations. With increasing human influences on the environment, one important challenge is to consider the role of plasticity in shaping organism responses to anthropogenic disturbances.

Chemical contaminants such as pesticides are widely applied in the environment to control pests and prevent human diseases. However, the evolution of pesticide resistance has become a major problem in controlling pest populations and today more than 500 pest species have evolved resistance to various chemicals (Georghiou 1990; Georghiou and Lagunes-Tejeda 1991). Considerable effort has sought to understand the mechanisms behind insecticide resistance, but this understanding has almost exclusively focused on targeted pest species (Jansen et al. 2011) and is strongly biased towards the assumption that resistance takes the form of a constitutive trait (Feyereisen 1995; Pimentel 2005; Lopes et al. 2008).

In contrast, the potential role of plasticity in shaping insecticide resistance (i.e., induced tolerance^1^) has rarely been considered. To our knowledge, only one other study has documented the induction of higher tolerance following prior exposure to pesticides. In that study, Poupardin et al. (2008) found that mosquitoes (*Aedes aegypti*) exposed to different contaminants could induce higher tolerance to several other contaminants, similar to how organisms exposed to predator cues subsequently become less susceptible to predators (Ferrari and Chivers 2009; Schoeppner and Relyea 2009). Given that exposure to insecticides can vary dramatically across space and time (Odenkirchen and Wente 2007), it seems reasonable that targeted pest species and nontargeted species may be able to respond to sublethal exposures by inducing higher tolerance to later exposures. If so, then induced tolerance could play a significant role in population persistence following an initial insecticide exposure. If inducible tolerance to pesticides occurs widely among taxa, it would alter our entire perspective on how pesticides affect organisms in nature.

There are three factors that could potentially affect the magnitude of induced insecticide tolerance: the concentration of the exposure, the timing of the exposure, and the history of a population's exposure. The concentration of the initial sublethal exposure should matter because it may affect an organism's ability to detoxify the insecticide. At low concentrations, researchers have shown that populations have evolved higher resistance by upregulating the enzymes that pesticides target, such as acetylcholine esterase (AChE; Georghiou 1990; Oakeshott et al. 2005). At higher concentrations, however, pesticides may overwhelm the ability for organisms to upregulate these enzymes or the cost of upregulation may simply be too high; if so, then we would not expect to observe induced tolerance at high pesticide concentrations. Given these predictions, it is important to examine the potential for induced tolerance across a range of concentrations.

The timing of the initial sublethal exposure during development should also affect induced tolerance. Toxicology research has found that lethal susceptibility to insecticides can vary with developmental stage (Bridges 2000; Aydın and Köprücü 2005; Jones et al. 2010), and plasticity research has found that developmental constraints (physiological, mechanical, energetic, etc.) commonly hinder an individual's ability to initiate plastic responses (Hensley 1993; Hoverman and Relyea 2007). Given the known importance of developmental windows of sensitivity to pesticides, examining the potential for induced tolerance in multiple developmental stages is critical.

The history of a population's exposure to pesticides is another factor that could affect induced tolerance. Plasticity theory predicts that when phenotypically plastic populations experience consistent inducing conditions over multiple generations, a plastic response can eventually become constitutively expressed, even in the absence of original cues that induced the phenotypic change (i.e., the process of genetic assimilation; Waddington 1942; Debat and David 2001). In contrast, when populations experience inconsistent conditions, inducible traits should be maintained because they facilitate the expression of adaptive phenotypes in response to variable environmental conditions (Schlichting and Pigliucci 1998). Applying this theory to induced tolerance, one would predict that induced tolerance would be more likely in populations that are not consistently exposed to insecticides, whereas constitutive tolerance would be more likely in populations that are consistently exposed to insecticides. Given these predictions, it is important to look for patterns of induced tolerance in multiple populations that differ in the regularity of their exposure to insecticides.

### Model system

Amphibians are an excellent model system to investigate the potential for inducible insecticide tolerance. Extensive research has demonstrated the ability of amphibians to alter their behavioral and morphological phenotypes in response to various stressors including competitors, predators, and pesticides (Relyea 2001, 2002, 2012; Van Buskirk 2009). Thus, amphibians may be able to respond to sublethal insecticide exposures, so it is reasonable to investigate whether insecticides can induce increased tolerance.

Given their well-described developmental stages (Gosner 1960), amphibians are also good models for investigating how exposure across ontogeny might affect the inducibility of tolerance. Previous work has shown that amphibian tolerance to insecticides varies across ontogeny, with embryos generally being more tolerant than tadpoles (Bridges 2000). The ability for amphibians to induce tolerance to stressors during more tolerant life stages can have significant lasting consequences on more susceptible life stages by decreasing the lag time between the exposure to a stressor and the ability to mount adaptive responses (Ferrari and Chivers 2009).

Amphibians are also an ideal system to investigate how the proximity of populations to agriculture might impact the ability for individuals to induce tolerance. For example, ponds <200 m from agriculture are not strongly affected by insecticides (Declerck et al. 2006), which means that amphibian populations that are closer to agricultural fields may be more commonly exposed to pesticides. In support of this pattern, Cothran et al. (2013) recently discovered that wood frog [*Rana sylvatica* (*Lithobates sylvaticus*)] populations living closer to agriculture are more tolerant to insecticides than populations living far from agriculture. Thus, it is likely that proximity to agriculture can contribute to shaping insecticide tolerance, and the distance from agriculture can provide a unique opportunity to investigate how amphibian populations exposed to different levels of agricultural activities including pesticide applications have evolved different magnitudes of induced tolerance.

Using larval wood frogs, our goal was to determine how different sublethal insecticide concentrations, different times of exposure in ontogeny (embryo and hatchling), and different populations close to and far from agriculture affect induced tolerance in wood frog tadpoles. Specifically, we tested these hypotheses: (i) exposure to sublethal pesticide concentrations will induce higher tolerance to the insecticide later in life, (ii) induced tolerance is more likely to occur when individuals are exposed to lower than higher sublethal concentrations, (iii) induced tolerance will occur in both the embryos and newly hatched tadpoles, (iv) populations located far from agricultural fields will exhibit greater induced tolerance than populations located close to agricultural fields, (v) tadpoles with induced tolerance will have higher AChE concentrations compared with noninduced tadpoles from the same population.

## Methods and materials

### Insecticide background

We chose to work with carbaryl (Sevin© 22.5% active ingredient; CAS 63-25-2), an AChE-inhibiting insecticide that currently dominates home insecticide sales and is also applied in agricultural settings for pest and malarial prevention (Grube et al. 2011). The half-life of carbaryl is 10 days at a pH of 7, and the maximum concentration detected aquatic systems is 33.5 ppb (U.S. EPA 2008). Carbaryl can enter amphibian habitats through direct application, drift, and run-off (Mitra et al. 2011) and operates by reversibly binding to AChE. With AChE inhibited, acetylcholine accumulates, leading to overstimulation of neurons and eventually mortality (Brown 2005; Lajmanovich et al. 2010). A well-established physiological mechanism of evolved tolerance in target pest species is the upregulation of AChE (Oakeshott et al. 2005). Thus, a possible mechanism by which amphibians might experience induced tolerance is by upregulating the production of AChE.

### Experimental design

**Figure 1:**
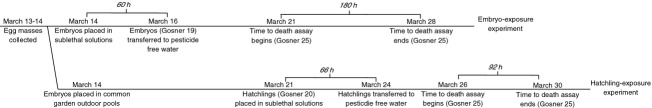
Experiment timeline.

To investigate the possibility of induced tolerance in wood frogs, we conducted an embryo-exposure experiment and a hatchling-exposure experiment (Fig. 1). Both experiments consisted of two distinct phases: Phase 1 consisted of an exposure to sublethal concentration of carbaryl to induce tolerance, whereas Phase 2 consisted of an exposure to a lethal concentration of carbaryl to quantify time to death (TTD) and assess tolerance. Time to death assays are useful tools for assessing relative tolerance among different groups and are also good indicators of an individual's insecticide tolerance (Bridges and Semlitsch 2000; Semlitsch et al. 2000).

To understand how population proximity to agriculture might impact the ability to induce tolerance, we conducted the embryo and hatchling experiments using individuals from four populations that vary in proximity to agriculture. Past studies have demonstrated that agricultural fields >200 m from small ponds do not have strong effects on aquatic systems (Declerck et al. 2006), thus we chose two populations from ponds that were close to agricultural fields (<100 m from the edge of the pond) and two populations that were far from agricultural fields (>800 m; Table S1). A recent study also demonstrated that tadpoles from these populations also vary in their tolerance to carbaryl (Hua et al. 2013). Tadpoles from populations far from agricultural fields (Hopscotch Pond and Square Pond) are more susceptible to carbaryl than tadpoles from populations close to agricultural fields (Trailer Park Pond and Staub Pond; Table S1). On 13–14 March 2012, we collected seven to 10 newly oviposited egg masses from each of the four wood frog populations. We immediately placed all collected egg masses into plastic buckets filled with ∼9 L of filtered water. Populations were kept separate but egg clutches from the same population were mixed together (pH = 7; Gosner stage 5).

### Embryo-exposure experiment

#### Phase 1 – sublethal exposure to induce tolerance

Immediately after eggs were collected, we isolated 800 individual embryos from each of the four populations (Gosner stage 5; Gosner 1960) by individually separating an equal number of embryos from each of the egg masses while keeping the jelly coat of each individual embryo intact. After separating the individuals from their egg masses, we distributed individual eggs into a completely randomized, factorial design with animals from the four populations crossed with four sublethal exposures (nominal concentrations: 0, 0.1, 0.5, or 1 ppm of carbaryl). This produced a total of 16 treatments, which we replicated four times each for a total of 64 experimental units. Our experimental units were 500 mL plastic containers filled with 450 mL of well water. From the 800 embryos representing each population, we randomly assigned groups of 200 embryos into each of the four sublethal carbaryl treatments. Each treatment was replicated four times (50 individuals/container). We reared the embryos in the laboratory at a constant temperature of 20°C. Pesticide solutions were not renewed. After being exposed for 60 h (Fig. 1), embryos reached prehatchling stage (Gosner stage 19). Keeping together individuals in each of the four replicates, tadpoles were transferred to 450 mL of pesticide-free, filtered well water until they hatched. On 17 March, again keeping individuals together by replicate, we transferred all hatchlings to 14-L containers filled with 7 L of pesticide-free well water. Hatchlings were held in clean water for 4 days prior to Phase 2 of the experiment (Gosner 25) and were not fed because they were still living off of yolk reserves.

All eggs not used in the embryo-exposure experiment were placed in 100-L outdoor pools containing 90 L of aged well water (outdoor air temperature ranged from 11 to 24°C). We used these eggs in the subsequent hatchling-exposure experiment.

#### Phase 2 – lethal exposure to assess induced tolerance

Prior to the start of Phase 2, we randomly chose 10 tadpoles from each of the 16 treatments and measured tadpole mass. To determine whether the initial exposure to sublethal concentrations of carbaryl induced tolerance later in life, we crossed the 16 treatments from Phase 1 with two subsequent carbaryl exposures in Phase 2. The two carbaryl treatments in Phase 2 were a no-pesticide control (i.e., water) and a concentration that should be lethal to tadpoles. Using a factorial, completely randomized design, this produced 32 treatments replicated five times each, for a total of 160 experimental units. The experimental units were 100-mL Petri dishes filled with either 70 mL of water or 70 mL of the lethal carbaryl solution.

When conducting TTD assays, the objective is to cause some mortality but not complete and immediate mortality (Newman 2010). For carbaryl, the estimated LC50 value for wood frogs is 1.2 ppm (Relyea 2003) and past studies found that wood frogs were highly susceptible to 30 ppm (Bridges and Semlitsch 2000). Based on these data, we chose an intermediate concentration of 8 ppm of carbaryl for the TTD assay.

On 21 March, we randomly added 10 tadpoles (Gosner stage 25) from each of the 16 embryonic treatments to either water or 8 ppm of carbaryl. To assess tadpole tolerance, we measured TTD and total survival. We assessed tadpole mortality every 4 h for 180 h and conducted a water change and reapplication of carbaryl every 24 h. Due to low mortality (<1%) after 72 h with 8 ppm of carbaryl, we increased the concentration to 15 ppm and continued to observe low mortality (<1%) after 96 h. As a result, we further increased the concentration to 18 ppm. These nominal carbaryl concentrations are high relative to what can be found in nature (Norris et al. 1983), but our objective was to discriminate among treatment groups in terms of pesticide tolerance to determine whether prior pesticide exposure led to increased levels of tolerance. We maintained the nominal concentration of 18 ppm for subsequent water changes for 180 h (7.5 days). After 180 h, we terminated the TTD assay and documented the number of surviving tadpoles. In accordance with standard toxicity tests, tadpoles were not fed during the test (ASTM 2008). The hatchling tadpoles had food reserves in the form of yolk as evidenced by the low mortality (1–2% among the four populations; Table S3) observed in animals exposed to 0 ppm of carbaryl in the TTD assay. All methods were approved by the University of Pittsburgh's IACUC (protocol 12050451).

### Hatchling-exposure experiment

To conduct the hatchling-exposure experiment, we followed the same two-phase experimental design as the embryo-exposure experiment using the remaining individuals from each population that we reared in outdoor pools. Because these embryos were reared outside with lower temperatures (but within the range of temperatures experienced in nature), they developed more slowly and this allowed us to stagger the embryo- and hatchling-exposure experiments while using animals from the same genetic backgrounds.

#### Phase 1 – sublethal exposure to induce tolerance

Using the same method as the embryo-exposure experiment, we took animals from the same four populations and crossed them with four sublethal exposures (nominal concentrations: 0, 0.1, 0.5, or 1 ppm of carbaryl). This produced a total of 16 treatments, which we replicated four times each for a total of 64 experimental units. Our experimental units were 14-L plastic containers filled with 7 L of one of the four of sublethal carbaryl solutions. We brought the embryos held outdoors (∼550 individuals from each population) back into the laboratory on 20 March. All embryos hatched indoors (Gosner stage 20) by 21 March, and we randomly assigned 128 hatchlings to each of the four sublethal carbaryl treatments (32 individuals/replicate). On 24 March, after being exposed to these sublethal concentrations of carbaryl for 66 h, keeping individuals together within replicates, we transferred all hatchlings (Gosner stage 24) to pesticide-free water for 2 days prior to Phase 2 (Fig. 1).

#### Phase 2 – lethal exposure to assess induced tolerance

Prior to the TTD assay (Phase 2), we randomly chose 10 tadpoles from each of the 16 treatments and measured tadpole mass. Using the same protocol as the embryonic experiment, we exposed tadpoles (Gosner stage 25) from each of the 16 hatchling treatments to either 0 or 15 ppm of carbaryl. The 32 treatments were replicated five times for a total of 160 experimental units (100-mL Petri dishes filled with 70 mL of 0 or 15 ppm of carbaryl). We assessed tadpole mortality every 4 h for 92 h and changed the Petri dish solutions every 24 h. At 92 h, we ended the experiment because tadpoles in the 0 ppm treatment of the TTD assay began to experience mortality, although mortality was low (1–11% among the four populations; Table S3). At 92 h, we documented final tadpole survival.

### Pesticide applications

To generate pesticide solutions, we created a stock solution by dissolving a commercial grade solution of carbaryl (Sevin^©^; Garden Tech, Lexington, KY, USA) in filtered water. To achieve the stock solutions of the four sublethal concentrations for Phase 1 (0.1, 0.5, and 1 ppm), we added 4, 7 and 15 μL of commercial grade carbaryl with 8.5, 3.5 and 3.5 L of filtered water, respectively. We then added 450 mL of these stock solutions to 500-mL experimental units. For the embryonic TTD assay (Phase 2), we created a stock solution to achieve 8, 15, and 18 ppm by adding 245, 460, and 552 μL of commercial grade carbaryl into 7.2 L of filtered water, respectively. Similarly, for the hatchling TTD assay, stock solutions were prepared by adding 460 μL to 7.2 L of filtered water to achieve 15 ppm. We then added 70 mL of these stock solutions to each of the Petri dish experimental units. Finally, for both experiments, we used filtered water to create the control stock solutions.

### Pesticide testing

To determine the actual concentrations of the pesticides used in this study, we collected samples (500 mL) of each stock solution immediately after animals were added in Phase 1 and after every water change in Phase 2. Samples were stored in amber jars and kept at 4°C in accordance with established analytical methods (OECD 2007). Samples were sent to an independent laboratory, and concentrations were tested using aqueous injection HPLC with postcolumn derivatization (Georgia Chemical Laboratory, Athens, GA, USA). As we used identical methodologies to generate pesticide solutions for both the embryonic and hatchling-exposure experiments, we only sent embryonic exposure experiment samples to be tested. Stock solutions were analyzed within 5 weeks of the sampling date, and actual concentrations for the 0.1, 0.5, and 1 ppm nominal concentrations were 0.07, 0.25, and 0.62 ppm of carbaryl. Actual concentrations for the lethal concentrations of 8, 15, and 18 ppm were 3.7, 6.2, and 7.1 ppm. Carbaryl was not detectable in our control treatments (minimum detectability = 0.01 ppm). Despite storing samples in accordance with established analytical methods (OECD 2007), the actual concentrations were lower than nominal concentrations likely due to sample degradation through a variety of biological and chemical processes (Sherma and Beroza 1980). When describing our results, we refer to the actual concentrations.

### Acetylcholine esterase assay

To determine whether any observations of induced tolerance were associated with increases in AChE expression, we measured the concentration of total tadpole AChE in a sample of tadpole bodies. For embryo- and hatchling-exposed tadpoles, we measured AChE concentration prior to Phase 2. In the hatchling-exposure experiment, we also measured the AChE concentration in the surviving tadpoles immediately after Phase 2. Following Phase 2 of the embryo-exposure experiment, several treatments had replicates where no tadpoles survived. Consequently, we were unable to assess AChE concentrations with equal representation from each replicate, thus we did not analyze AChE concentration after Phase 2 for the embryonic-exposure experiment.

To quantify AChE concentrations, we subsampled 10 individuals from each treatment by haphazardly selecting two individuals from each of the five replicates from Phase 2. We individually homogenized whole tadpoles with 10% octylphenoxypolyethoxyethanol (triton X-100) in 0.1 m tris (hydroxymethyl) aminomethane hydrochloride (pH = 7.4) using the Ellman method (1961). The homogenates were centrifuged at 23 447 G for 15 min. AChE concentration of each tadpole was determined using the Ellman colorimetric method (Ellman et al. 1961); these 10 measurements were then averaged by treatment. The reaction mixture consisted of 200 μL of tadpole homogenate, 50 μL of 20 mm dithiobis 2-nitrobenzoic acid (DTNB), and 50 μL of 20 mm acetylthiocholine (AcSCh) in 96-well plates. After a 15-min incubation period at 20°C, each sample was assayed in duplicate by measuring absorbance at 405 nm using an Epoch 96-well plate spectrophotometer.

### Statistical analysis

To investigate the possibility of induced tolerance, we compared rates of tadpole survival in the TTD assay when previously exposed to different sublethal insecticide concentrations. We analyzed the data from the embryonic and hatchling experiment using a separate Cox's proportional hazards model for each population (Cox 1972). Using this method of survival analysis, we used the TTD of each individual tadpole to determine hazard ratios, which examine the probability of mortality of animals previously exposed to various sublethal carbaryl concentrations in Phase 1 relative to animals exposed to 0 ppm in Phase 1. A hazard ratio <0 for tadpoles that were initially exposed to insecticides as embryos or hatchlings indicates a decrease in the probability of mortality compared with individuals exposed to no insecticides as embryos or hatchlings. In contrast, a hazard ratio <0 for tadpoles that were initially exposed to insecticides as embryos or hatchlings indicates an increase in the probability of mortality compared with individuals exposed to no insecticides as embryos or hatchlings. We define induced tolerance as cases where treatment hazard ratios are significantly lower than the control (hazard ratio = 0).

Following the TTD assay, we also analyzed survival of tadpoles in each experimental unit (Petri dish) using an anova. Significant univariate results were assessed using Tukey's pairwise comparison test, and significant interactions were analyzed using targeted post hoc tests that separately compared the effect of each sublethal concentration within a population. Although less sensitive, the survival results were consistent with TTD results. Thus, we do not present and discuss the anova results for survival in the main text (but see Appendix S1, Figure S1, Figure S2, and Table S4).

For the embryo-exposure experiment, we analyzed the AChE concentration of tadpoles prior to the TTD assay using an anova. Significant univariate results were assessed using Tukey's pairwise comparison test, and significant interactions were analyzed using targeted *post hoc* tests that separately compared the effect of each sublethal concentration for each population. Because we subsampled 10 tadpoles from five replicates and did not track which tadpoles came from each replicate, we could not take the mean AChE from each replicate. Instead, we had to analyze the data based on the 10 samples from each treatment. To guard against inflated degrees of freedom associated with this pseudoreplication, we conducted the analysis using the degrees of freedom based on the number of independent replicate containers (i.e., five) rather than on the number of tadpoles actually measured (i.e., 10).

For the hatchling-exposure experiment, we analyzed the AChE concentration of tadpoles before and after Phase 2 using separate anovas. We did not use a repeated-measures analysis because different individuals were measured before and after Phase 2. We conducted these analyses in the same manner as the embryo-exposure experiment.

Finally, we used an anova to confirm that there were no differences in mass by comparing mass of tadpoles prior to Phase 2 in both the embryo- and hatchling-exposure experiments.

## Results

### Time to death in Phase 2: embryo-exposure experiment

**Figure 2:**
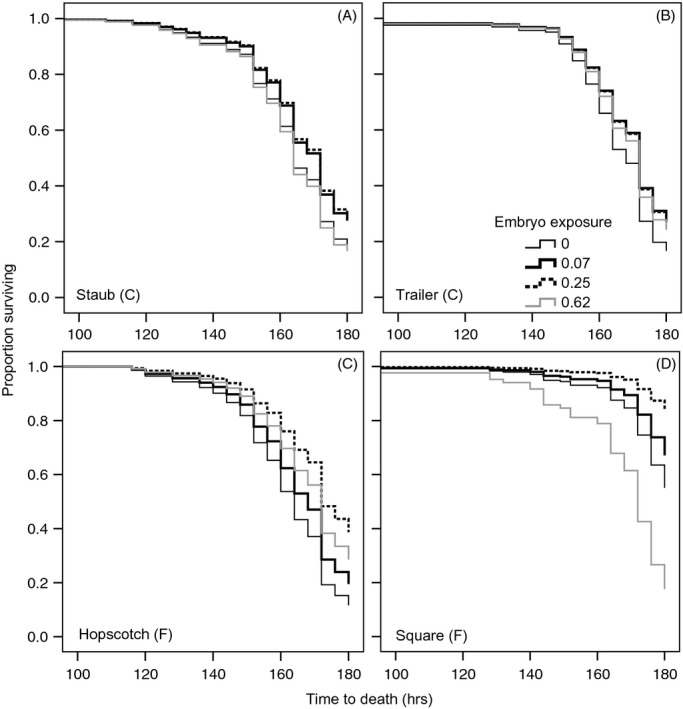
Survival across time of tadpoles exposed to lethal carbaryl as embryos. The abbreviation ‘F’ indicates populations far from agricultural fields (>800 m), whereas ‘C’ indicates populations close to agricultural fields (<100 m).

We found that embryonic exposure to sublethal carbaryl concentrations during Phase 1 did not affect the mass of tadpoles prior to Phase 2 (*F*_3,144_ = 1.1; *P* = 0.38). We also found high survival in the control treatment of the TTD assay (Average ± SE; Hopscotch = 96% ± 1.5; Square = 99% ± 1; Staub = 96% ± 2.1; Trailer = 98% ± 0.99). To assess TTD in the lethal treatments, we used the Cox proportional hazard test to determine whether the initial exposure to carbaryl as embryos in Phase 1 affected the subsequent mortality of tadpoles exposed to a lethal concentration of carbaryl in Phase 2. For tadpoles, from populations that are more tolerant and located close to agriculture (Staub Pond and Trailer Park Pond), the initial exposure to carbaryl had no effect on their tolerance to carbaryl later in life (Table 1; Fig. 2A,B). In contrast, tadpoles from populations that are less tolerant and located far from agriculture (Hopscotch Pond and Square Pond) experienced increased tolerance to carbaryl later in life (Fig. 2C,D). For Hopscotch Pond, tadpoles that were exposed to the highest sublethal concentrations as embryos (i.e., 0.25 and 0.62 ppm of carbaryl) became more tolerant to a lethal concentration of carbaryl (significantly lower hazard ratio) than tadpoles that were not exposed to carbaryl as embryos. For Square Pond, tadpoles exposed to 0.25 ppm of carbaryl as embryos became more tolerant to a lethal concentration of carbaryl than tadpoles not exposed as embryos. However, tadpoles exposed to 0.62 ppm of carbaryl as embryos became less tolerant to a lethal dose of carbaryl than tadpoles not exposed as embryos.

**Table 1:** Hazard ratios for tadpoles from four populations had been previously exposed to four sublethal concentrations of carbaryl as embryos (0, 0.1, 0.5, or 1.0 ppm) and then re-exposed as tadpoles to a lethal concentration of carbaryl (18 ppm). Negative hazard ratios indicate that the initial exposure made tadpole more tolerant, whereas positive hazard ratios indicate that the initial exposure made tadpole less tolerant

	Hazard ratios for the initial carbaryl exposures as embryos (*P*-value)			
		
Population	0.07 ppm	0.25 ppm	0.62 ppm	Percentage censored
Hopscotch	−0.28 (0.21)	−0.82 **(<0.001)**	−0.54 **(0.019)**	25
Square	−0.4 (0.22)	−1.2 **(0.003)**	1.07 **(<0.001)**	57
Staub	−0.27 (0.25)	−0.31 (0.18)	0.06 (0.77)	23
Trailer Park	−0.33 (0.16)	−0.32 (0.17)	−0.24 (0.30)	24

Bold values represent significant *p*-values < 0.05.

### Time to death in Phase 2: hatchling-exposure experiment

**Figure 3:**
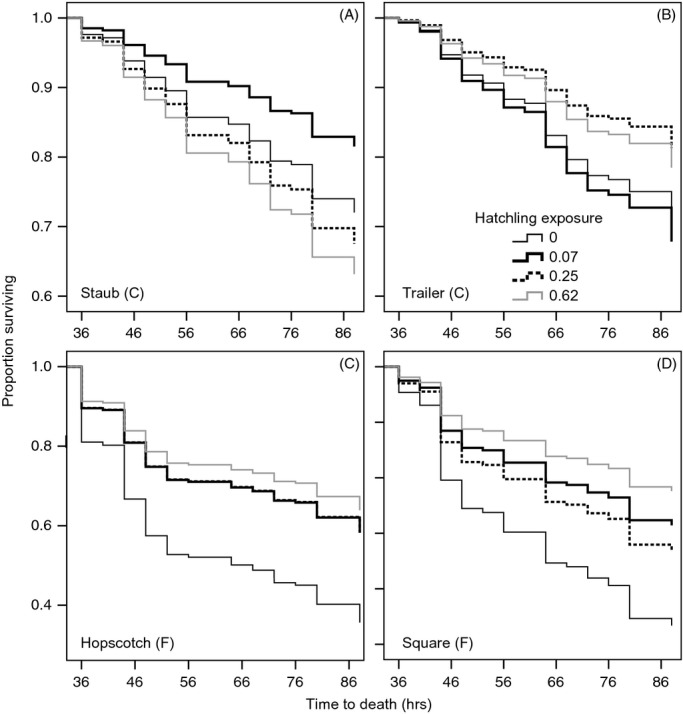
Survival across time of tadpoles exposed to lethal carbaryl as hatchlings. The abbreviation ‘F’ indicates populations far from agricultural fields (>800 m), whereas ‘C’ indicates populations close to agricultural fields (<100 m).

Hatchling exposure to sublethal carbaryl concentrations during Phase 1 did not affect tadpole mass prior to Phase 2 (*F*_3,144_ = 0.88; *P* = 0.45). We also found high survival in the control treatment of the TTD assay (Average ± SE; Hopscotch = 73% ± 4.5; Square = 90% ± 3.1; Staub = 92% ± 3.5; Trailer = 96% ± 1.9). When we conducted the Cox proportional hazard test, we found that the initial exposure of hatchlings to sublethal carbaryl had no subsequent effect on the mortality of tadpoles from populations located close to agriculture (Staub and Trailer Park Ponds) when exposed to lethal carbaryl concentrations (Table 2; Fig. 3A,B). In contrast, tadpoles from the populations located far from agriculture (Hopscotch and Square Ponds) exhibited increased tolerance when exposed to lethal carbaryl concentrations. For Hopscotch Pond, tadpoles that were initially exposed to 0.07, 0.25, and 0.62 ppm of carbaryl as hatchlings became more tolerant to the subsequent lethal carbaryl concentration than tadpoles that were not initially exposed (Table 2; Fig. 3C,D). For Square Pond, only tadpoles exposed to 0.62 ppm as hatchlings were more tolerant to a lethal concentration of carbaryl than tadpoles that were not exposed to carbaryl as hatchlings.

**Table 2:** Hazard ratios for tadpoles from four populations had been previously exposed to four sublethal concentrations of carbaryl as hatchlings (0, 0.1, 0.5, or 1.0 ppm) and then re-exposed as tadpoles to a lethal concentration of carbaryl (15 ppm). Negative hazard ratios indicate that the initial exposure made tadpole more tolerant, whereas positive hazard ratios indicate that the initial exposure made tadpole less tolerant

	Hazard ratios for the initial carbaryl exposures as hatchlings (*P*-value)			
		
Population	0.07 ppm	0.25 ppm	0.62 ppm	Percentage censored
Hopscotch	−0.64 **(0.023)**	−0.65 **(0.024)**	−0.83 **(0.004)**	55
Square	−0.62 (0.06)	−0.45 (0.17)	−0.91 **(0.013)**	68
Staub	−0.47 (0.27)	0.18 (0.63)	0.34 (0.34)	71
Trailer Park	0.1 (0.77)	−0.53 (0.21)	−0.37 (0.36)	74.5

Bold values represent significant *p*-values < 0.05.

### Acetylcholine esterase concentrations: embryo-exposure experiment

**Figure 4:**
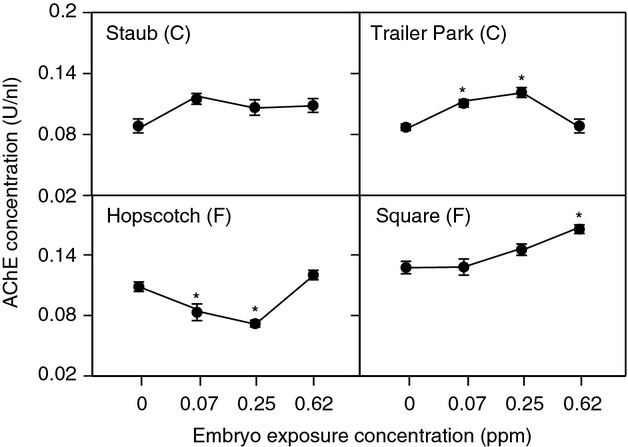
Acetylcholine esterase concentrations of tadpoles exposed to sublethal concentrations of carbaryl as embryos before Phase 2. Asterisks represent significant differences (*P* < 0.05) in tadpole AChE concentration between hatchlings that were not initially exposed to carbaryl as embryos and hatchlings that were initially exposed to carbaryl.

For the embryo-exposure experiment, we assessed AChE concentrations of all four populations before Phase 2 and found significant effects of population, concentration, and their interaction (Table 3A). Post hoc comparisons indicated a significant effect of sublethal pesticide exposure on tadpoles from Hopscotch (far), Square (far), and Trailer Park Pond (close), but not on Staub Pond (close; Table 3B). Additionally, tadpoles from Trailer Park pond that were initially exposed to intermediate carbaryl concentrations (0.07 and 0.25 ppm) had significantly higher AChE concentrations than the control (Fig. 4). Tadpoles from Hopscotch Pond exposed to 0.07 and 0.25 ppm of carbaryl had significantly lower AChE concentrations than the control (Fig. 4). Tadpoles from Square Pond that were initially exposed to the highest concentration of carbaryl (0.62 ppm) had higher AChE concentrations than the control.

**Table 3:** (A) Test results from anovas on AChE concentrations in tadpoles (1) before Phase 2 in the embryo-exposure experiment, (2) before Phase 2 in the hatchling exposure, and (3) after Phase 2 in the embryo-exposure experiment. (B) AChE concentrations anova by population from tadpoles: (1) before Phase 2 in the embryo-exposure experiment and (2) before Phase 2 in the hatchling-exposure experiment

A. AChE concentration anova results	df	*F*	*P*-value
1. Embryo exposure, before Phase 2			
Population	3, 48	14.7	**<0.001**
Concentration	3, 48	7.0	**<0.001**
Pop'n × Conc	9, 48	10.8	**<0.001**
2. Hatchling exposure, before Phase 2			
Population	3, 48	5.62	**0.003**
Concentration	3, 48	0.57	0.64
Pop'n × Conc	9, 48	2.101	**0.047**
3. Hatchling exposure, after Phase 2			
Population	3, 64	3.1	**0.03**
Concentration	3, 64	9.7	**<0.001**
Pop'n × Conc	9, 64	0.98	0.38

(B) AChE concentrations anova by population from tadpoles				
1. Embryo exposure, before Phase 2	Hopscotch pond (df = 3, 12)	Square pond (df = 3, 12)	Trailer pond (df = 3, 12)	Staub pond (df = 3, 12)
Pesticide	**<0.001**	**<0.001**	**<0.001**	0.17
2. Hatchling exposure, before Phase 2	Hopscotch pond (df = 3, 12)	Square pond (df = 3, 12)	Trailer pond (df = 3, 12)	Staub pond (df = 3, 12)
Pesticide	0.18	0.20	0.06	0.43

Bold values represent significant *p*-values < 0.05.

### Acetylcholine esterase concentrations: hatchling-exposure experiment

**Figure 5:**
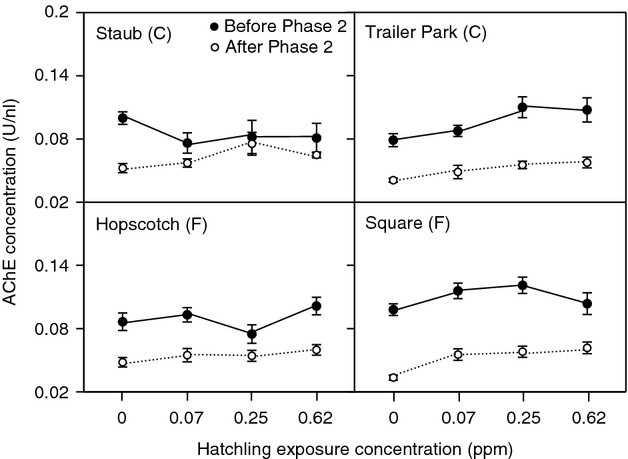
Acetylcholine esterase concentration of tadpoles exposed to sublethal concentrations of carbaryl before and after Phase 2. Closed circles represent AChE concentrations of tadpoles measured before Phase 2, and opened circles represent AChE concentrations measured after Phase 2.

For the hatchling-exposure experiment, we assessed AChE concentrations on tadpoles that had been initially exposed to sublethal carbaryl concentrations before Phase 2 and again on tadpoles that survived Phase 2. In the first analysis, we found an effect of population and a population-by-concentration interaction (Table 3A). Our post hoc comparisons found no significant effects of sublethal carbaryl exposure concentrations within each of the populations (Table 3B; Fig. 5).

In the analysis of tadpoles that survived Phase 2, we found a significant effect of population and concentration but no interaction (Table 3A). Our post hoc comparisons found no differences among the four populations. In contrast, of the tadpoles that survived the TTD assay, those that were exposed to sublethal concentrations of carbaryl as hatchlings had significantly higher AChE concentrations compared with tadpoles not exposed to carbaryl as hatchlings.

## Discussion

We discovered that exposing amphibian embryos and hatchlings to sublethal concentrations of carbaryl can induce increased tolerance later in life. Interestingly, the pattern of induced tolerance only emerged in tadpoles from populations that were far from agricultural fields. For both far from agriculture populations, insecticide concentration was important to predicting patterns of induced tolerance. Generally, for both the embryo and hatchling experiments, as the concentration of the initial sublethal application increased, the magnitude of induced tolerance also increased. However, this was not the case for Square Pond (far); tadpoles exposed to the highest sublethal concentration (0.62 ppm) as embryos became less tolerant to a subsequent lethal exposure of carbaryl. In our assessment of AChE, we found that exposure to sublethal concentrations both significantly increased and decreased tadpole AChE concentrations.

Plasticity theory predicts that populations that experience variable environmental conditions should maintain the ability to induce adaptive phenotypes in response to changing environments (Schlichting and Pigliucci 1998). In contrast, less variable environments should select for constitutive expression of traits and decreased plasticity. Consistent with this theory, we only observed induced tolerance in populations that were far from agriculture and would be unlikely to consistently experience insecticides (ponds <200 m from agriculture are not strongly affected by insecticides; Declerck et al. 2006). These populations might occasionally experience insecticides, such as those sprayed in forests to control tree-damaging insects, but such applications would not be as consistent over the years as insecticide applications on agricultural crops. Considerable effort has sought to understand insecticide resistance that arises via constitutive genetic pathways (i.e., evolved resistance; Lopes et al. 2008), but we demonstrate that for populations that experience less consistent insecticide exposures, induced tolerance may be an important alternative mechanism in which evolved resistance can be achieved, which suggests the process of genetic assimilation. An important next step is to determine the generalizability of this pattern across a broader agricultural gradient. Further, despite higher constitutive tolerances of populations close to agriculture, the lack of a plastic response suggests that there may be costs to maintaining constitutive resistance (Callahan et al. 2008). Future studies investing these potential costs are critical to understanding the relative contribution of evolved resistance versus induced tolerance in natural systems exposed to insecticides.

To our knowledge, this is the first study in vertebrates and only the second study among all animals to demonstrate that sublethal and ecologically relevant concentrations of a common insecticide can induce increased tolerance. The only other study was that of Poupardin et al. (2008), who exposed mosquito larvae for 24 h to sublethal concentrations of two insecticides (permethrin and temephos) and found that pre-exposed larvae subsequently had increased tolerance. Poupardin et al. (2012) went on to show that the insecticides select for different genes in individuals previously exposed to insecticides compared with those not previously exposed. Thus, the authors suggest that the ability to detect and induce responses to insecticides can have long-term impacts on how insecticides select for resistance. Future studies should investigate the contribution of induced tolerance to shaping the evolution of resistance.

We also found that the concentration of the initial pesticide exposure affected the patterns of induced tolerance. Generally, only the intermediate (0.25 ppm) and highest (0.62 ppm) sublethal concentrations resulted in induced tolerance. For both embryonic and hatchling experiments, there was only one case [tadpoles from Hopscotch (far) that were exposed as hatchlings] where exposure to the lowest sublethal concentration of carbaryl (0.07 ppm) led to induced tolerance. Thus, similar to threshold limits necessary for plastic responses of prey to predator cues (Relyea 2004; McCoy et al. 2012), a threshold insecticide concentration must also be met in order for the induction of tolerance. In nature, aquatic systems are often commonly exposed to low concentrations of insecticides (<1 ppm). Although low concentrations of chemicals are often not lethal to many taxa, the induction of tolerance following exposure to these low concentrations could potentially play a significant role in understanding population persistence following insecticide contamination.

Although we generally found that sublethal concentrations of insecticides induced increased tolerance, exposure of embryos from Square Pond (far) to the highest sublethal concentration of carbaryl induced a decrease in tolerance. For this population, we found that sublethal concentrations initiated a nonlinear hormetic dose response. A hormetic dose response is commonly found in response to environmental toxins (Costantini et al. 2010) and occurs when exposure to a high concentration of a chemical agent or environmental factor is damaging to an individual but lower exposures are beneficial to the individual compared with a control (Hayes et al. 2002; Mattson 2008). The results for Square Pond (far) indicate that the inducibility of tolerance for certain populations may be confined to a narrow range of concentrations. To understand the contribution of induced tolerance in natural systems, an important challenge is to identify the concentration windows in which induced tolerance is increased versus decreased.

Ontogenetic variation in insecticide tolerance is a broadly documented pattern, and we expected the ability to induce tolerance would vary across ontogeny. However, we found that embryos and hatchlings were both capable of experiencing induced tolerance. The ability for individuals to induce tolerance in early life stages may have significant lasting consequences in later life stages by decreasing the lag time between the exposure to a contaminant and the ability to mount adaptive responses (Ferrari and Chivers 2009). The majority of toxicological studies focus on assessing tolerance at single life stages, and few studies have considered how insecticide exposure early in development may increase future susceptibility (Bridges 2000; Jones et al. 2010). Amphibians are currently experiencing worldwide population declines for a variety of hypothesized reasons including exposure to insecticides (Sparling and Fellers 2007). The ability to induce tolerance to insecticides during more tolerant stages of ontogeny may have significant conservation implications for amphibians exposed to insecticides during more susceptible life stages.

In our assessment of AChE concentrations, we found that exposure to sublethal concentrations of carbaryl early in development can increase AChE concentrations in tadpoles. This pattern was especially prevalent for tadpoles that survived the TTD assay. Indeed, for the 12 treatments where tadpoles were exposed to sublethal concentrations of carbaryl as hatchlings, we found a significant increase in AChE concentration compared with tadpoles not exposed to sublethal carbaryl. A well-established physiological mechanism of tolerance in pest species is the upregulation of AChE (Oakeshott et al. 2005). Thus, a possible mechanism in which amphibians might experience induced tolerance is by upregulating AChE. Consistent with our hypothesis, the most tolerant individuals (survivors of TTD assay) from populations far from agriculture (Square and Hopscotch) in the hatchling experiment had significantly more AChE compared with the animals from the control treatment.

In contrast, for both embryo and hatchling experiments, when we measured AChE concentrations of tadpoles prior to the TTD assay, changes in AChE concentrations were less prevalent. Of the 24 total cases where embryos or hatchlings were exposed to sublethal carbaryl, we only found five cases (all in the embryo-exposed experiment) where sublethal exposure to carbaryl caused a significant change in AChE concentrations relative to the control. These data suggest that the pattern of increased AChE may be detectible only in the most tolerant individuals. In addition to being less prevalent, of the five cases where AChE concentrations were significantly affected, we found that the patterns of AChE concentration were not consistent with our predictions of induced tolerance. For three of these cases, sublethal concentrations of carbaryl caused an increase in AChE concentrations but there was no evidence of induced tolerance. For the other two cases, sublethal concentrations of carbaryl caused decreased AChE concentrations. Although we show that early exposure to sublethal concentrations of carbaryl can induce changes in AChE concentrations at later life stages, it is also possible that AChE may not be the only mechanism that allows for induced tolerance. Future studies are needed to identifying the relative contribution of AChE to induced tolerance.

## Conclusions

In rapidly changing environmental conditions, the contribution of plasticity has critical implications for individuals and the evolution of populations by allowing adaptive traits to be induced rapidly within a single generation. We are the first to demonstrate that sublethal and ecologically relevant concentrations of a common insecticide can, within the same generation, induce adaptive tolerance in amphibians. Understanding the role of induced tolerance can have significant conservation implications for populations of nontarget species exposed insecticide contaminants. Specifically, the pattern of inducibility varies by population but appears to be consistent with plasticity theory. Patterns of induced tolerance are dependent on insecticide concentration and occur at very early life stages. Finally, in our analysis of AChE concentrations, we find that exposure to sublethal concentrations has a lasting legacy on tadpole AChE concentrations. To sum, inducible responses to anthropogenic disturbances may have a significant impact on shaping patterns of species abundance. Future work identifying the underlying mechanisms that drive these inducible responses to insecticides as well as other anthropogenic contaminants are an important step towards understanding the long-term impact of disturbances on natural systems.

## Footnotes

1 We note that the distinction between the terms resistance and tolerance can vary across disciplines. Many toxicologists define tolerance as differences in susceptibility among species or life stages and resistance as the microevolution of decreased susceptibility over time. In this study, we focus on assessing variation in tolerance.

## Data archiving statement

Raw data of the main results are available in DRYAD: doi: 10.5061/dryad.5p8k0.

## Supporting Information

**Appendix S1**. Results from the analyses of variance

**Table S1**. Background information on each of the four populations.

**Table S2**. Tadpole tolerance to carbaryl (time to death) from populations close and far from agriculture in the absence of a previous sublethal exposure to carbaryl.

**Table S3.** Survival of tadpoles exposed to 0 ppm of carbaryl in the TTD assay for the Embryo-exposure and hatchling-exposure experiments.

**Table S4.** Test results from anovas on survival of tadpoles exposed to the lethal concentration during the TTD assay for A) the embryo-exposure experiment, B) the hatchling exposure experiment.

**Figure S1.** Survival of tadpoles exposed to four sublethal concentrations of carbaryl as embryos (0, 0.07, 0.25, and 0.62 ppm) followed by an exposure to a lethal concentration as tadpoles during the TTD assay.

**Figure S2.** Survival of tadpoles exposed to four sublethal concentrations of carbaryl as hatchlings (0, 0.07, 0.25, and 0.62 ppm) followed by an exposure to a lethal concentration as tadpoles during the TTD assay.
