# Supplementary material for: Pesticide tolerance in amphibians: induced tolerance in susceptible populations, constitutive tolerance in tolerant populations
Source: Evol Appl. 2013 Jul 25;6(7):1028–40. doi: 10.1111/eva.12083 (PMC3804236; doi:10.1111/eva.12083)
Supplement: Supplementary file 1 [file eva0006-1028-SD1.doc]

**Supplementary Information: Additional methods, results, tables, and figures.**

**Appendix S1. Results from the analyses of variance**

***Constitutive tolerance of tadpoles from the four populations***

To determine the constitutive tolerance of the four populations, we conducted a univariate analysis on the TTD of tadpoles exposed to 0 ppb in Phase 1 and the lethal concentration of carbaryl in Phase 2. Since these tadpoles were not subjected to insecticides in Phase 1, these TTD data provide an estimate of constitutive tadpole tolerances from each of the populations. Since tadpoles from the embryo and hatchling experiment faced different rearing conditions, we conducted separate ANOVAs for each experiment.

For the embryo experiment, we found that tadpole TTD did not differ between populations close to agriculture and populations far from agriculture (F_1, 18_ = 0.136; p = 0.71). However, tadpoles from populations close to agriculture tended to survive longer than those far from agriculture (Table S2). For the hatchling experiment, we found that tadpole TTD was higher for populations close to agriculture compared to populations far from agriculture (F_1, 18_ = 4.5; p = 0.049).

Based on these analyses, we conclude that tadpoles from populations close to agriculture were more tolerant to carbaryl than populations far from agriculture but this higher tolerance was only observed in the hatchling experiment. Tadpoles in both the embryo and hatchling-exposure experiment consisted of individuals from the same mixture of egg masses but tadpoles from these experiments varied in their rearing condition. Tadpoles from the embryo-exposure experiment were held indoors at a constant temperature of 20°C throughout the duration of the experiment while tadpoles from the hatchling were temporarily held outdoors for 7 d (Fig. 1) where outdoor temperatures fluctuated between 11 to 24°C. Thus, it is possible that the variation in rearing condition could have accounted for the variation in standing tadpole tolerance in the embryo and hatchling exposure experiments. Overall, though not always significant, populations close to agriculture had higher constitutive tolerance compared to populations far from (Table S2).

***Survival of tadpoles during the TTD after being exposed to carbaryl as embryos***

In the analysis of tadpole survival during the TTD assay, we found a significant effect of population, concentration, and a population-by-concentration interaction (Table S3). Because of the interaction, we ran separate ANOVAs for each population. The univariate ANOVA demonstrated that embryonic exposure to carbaryl only affected the survival of tadpoles from the more susceptible populations that were also farther from agriculture (i.e. Hopscotch and Square Pond; Table S2). Tadpoles from Hopscotch pond experienced lower mortality if they had been previously exposed to 0.5 or 1 ppm of carbaryl as embryos (F_3,16_ = 3.5; P = 0.04). In contrast, tadpoles from Square pond experienced higher mortality if they had been previously exposed to 1 ppm of carbaryl as embryos (Fig. S1; F_3,16_ = 13.9; P < 0.001).

***Survival of tadpoles during the TTD after being exposed to carbaryl as hatchlings***

In the analysis of tadpole survival during the TTD assay, we found a significant effect of population, but no effect of concentration or a population-by-concentration interaction (Table S3). When we conducted a Tukey’s pairwise test, we found that Square Pond was significantly different from Hopscotch (p = 0.002), Staub (p = 0.001), and Trailer park (p = 0.002) ponds.

Table S1. Background information on each of the four populations.

| **Population** | **Percent agriculture**  **(200 m radius)** | **Distance to closest agriculture** | **Tadpole tolerance^1^** | **GPS Location** |
| --- | --- | --- | --- | --- |
| Staub | 18.6 % | 97 m | 74.4 ± 2.6 | 41.59° N, 80.43° W |
| Trailer Park | 9.5 % | 62 m | 77.2 ± 2.5 | 41.57° N, 80.45° W |
| Hopscotch | 0 % | 879 m | 64.0 ± 1.9 | 41.87° N, 80.47° W |
| Square | 0 % | 1264 m | 58.8 ± 2.2 | 41.84° N, 80.24° W |

1. Mean time to death (hrs) ± SE of tadpoles following exposure to 6 ppm of carbaryl are based on the experiments of Hua et al. (2013).

S2. Tadpole tolerance to carbaryl (time to death) from populations close and far from agriculture in the absence of a previous sublethal exposure to carbaryl.

| **Proximity to agriculture** | **Embryo-experiment** | **Hatchling-experiment** |
| --- | --- | --- |
| Far | 138.84 ± 10.9 hrs | 42.5 ± 7.3 hrs |
| Close | 146.2 ± 4.5 hrs | 62.4 ± 3.3 hrs |

Table S3. Survival of tadpoles exposed to 0 ppm of carbaryl in the TTD assay for the Embryo-exposure and hatchling-exposure experiments.

| Survival in TTD assay of control treatments | | | | Phase 2 |  | | | | | |
| --- | --- | --- | --- | --- | --- | --- | --- | --- | --- | --- |
|  |  |  |  | Embryo-exposure | | | | Hatchling-exposure |  | |
| Proximity to agriculture | Population | Phase 1 treatment | Control survival (%) | | | SE | Control survival (%) | | | SE |
| Far | Hopscotch | 0 | 98 | | | 2 | 84 | | | 11.2 |
|  |  | 0.1 | 96 | | | 4 | 90 | | | 7.7 |
|  |  | 0.5 | 98 | | | 2 | 90 | | | 6.3 |
|  |  | 1 | 98 | | | 2 | 92 | | | 5.8 |
|  | Square | 0 | 100 | | | 0 | 96 | | | 2.4 |
|  |  | 0.1 | 100 | | | 0 | 92 | | | 8 |
|  |  | 0.5 | 96 | | | 4 | 96 | | | 2.4 |
|  |  | 1 | 100 | | | 0 | 98 | | | 2 |
| Close | Staub | 0 | 100 | | | 0 | 100 | | | 0 |
|  |  | 0.1 | 94 | | | 4 | 98 | | | 2 |
|  |  | 0.5 | 100 | | | 0 | 96 | | | 2.4 |
|  |  | 1 | 98 | | | 2 | 86 | | | 14 |
|  | Trailer park | 0 | 100 | | | 0 | 98 | | | 2 |
|  |  | 0.1 | 100 | | | 0 | 100 | | | 0 |
|  |  | 0.5 | 98 | | | 2 | 100 | | | 0 |
|  |  | 1 | 98 | | | 2 | 100 | | | 0 |

Table S4. Test results from ANOVAs on survival of tadpoles exposed to the lethal concentration during the TTD assay for A) the embryo-exposure experiment, B) the hatchling exposure experiment.

|  | | df | | F | | *P*-value |  |
| --- | --- | --- | --- | --- | --- | --- | --- |
|  | Population | 3, 64 | 7.6 | | **<0.001** | | |
| A. Embryo-exposure experiment | Concentration | 3, 64 | 3.4 | | **0.022** | | |
|  | Pop’n x Conc | 9, 64 | 2.2 | | **0.036** | | |
|  | Population | 3, 64 | 7.96 | | **<0.001** | | |
| B. Hatchling-exposure experiment | Concentration | 3, 64 | 0.45 | | 0.72 | | |
|  | Pop’n x Conc | 9, 64 | 0.59 | | 0.79 | | |

**Supplementary Information figure legends**

Figure S1. Survival of tadpoles exposed to four sublethal concentrations of carbaryl as embryos (0, 0.07, 0.25, and 0.62 ppm) followed by an exposure to a lethal concentration as tadpoles during the TTD assay.

Figure S2. Survival of tadpoles exposed to four sublethal concentrations of carbaryl as hatchlings (0, 0.07, 0.25, and 0.62 ppm) followed by an exposure to a lethal concentration as tadpoles during the TTD assay.

Figure S1.

Figure S2.
